# The Associations between Sibling Victimization, Sibling Bullying, Parental Acceptance–Rejection, and School Bullying

**DOI:** 10.3390/ijerph192316346

**Published:** 2022-12-06

**Authors:** Aiche Sabah, Musheer A. Aljaberi, Chung-Ying Lin, Hsin-Pao Chen

**Affiliations:** 1 Faculty of Human and Social Sciences, Hassiba Benbouali University of Chlef, Chlef 02076, Algeria; 2 Faculty of Medicine and Health Sciences, Taiz University, Taiz 6803, Yemen; 3 Department of Community Health, Faculty of Medicine and Health Sciences, Universiti Putra Malaysia, Serdang 43300, Malaysia; 4 Faculty of Nursing and Applied Sciences, Lincoln University College, Petaling Jaya 47301, Malaysia; 5 Institute of Allied Health Sciences, College of Medicine, National Cheng Kung University, Tainan 701, Taiwan; 6 Division of Colon and Rectal Surgery, Department of Surgery, E-DA Hospital, School of Medicine, College of Medicine, I-Shou University, Kaohsiung 824, Taiwan

**Keywords:** sibling bullying, school bullying, adolescent students, parental acceptance and rejection, violence, children’s health

## Abstract

Bullying has been identified as the most common form of aggression experienced by school-age youth. However, it is still unclear about the family’s influence on school bullying. Therefore, the current study aimed to explore the associations between sibling bullying and school bullying, sibling victimization and school victimization, and parental acceptance–rejection and school bullying victimization. The study was cross-sectional and conducted on a sample of students aged between 11 and 20 years recruited from middle schools in Algeria. The study used a survey adopted from the scale of Sibling Bullying, Student Survey of Bullying Behavior—Revised 2, and the Survey of parental acceptance–rejection in collecting the data. The model’s results assessing the association between sibling bullying and school bullying demonstrated that the effect of sibling physical and sibling verbal victims on school victimization was statistically significant. Despite the non-significant effect of sibling emotional victims on school victimization, the effect of sibling physical and sibling verbal bullying on school bullying was statistically significant. However, the effect of sibling emotional bullying on school bullying was not statistically significant. The direct effect of parental acceptance on school victimization was not statistically significant, whereas the effect of parental rejection on school victimization was statistically significant. The direct effect of parental acceptance on school bullying was not statistically significant, while the effect of parental rejection on school bullying was statistically significant. Based on the results, this study provides insights into the understanding of how the family and siblings contribute to school bullying. In particular, sibling victimization, sibling bullying, and parental acceptance–rejection are predictive factors of school bullying among adolescents. Future research should take into account factors based on family to explore the risks of school bullying.

## 1. Introduction

School violence, a type of violence that happens in school, including different subtypes (e.g., bullying) to cause physical and psychological harm to students, has attracted attention in the literature [1,2,3]. Although bullying is a subtype of school violence, bullying has been further found to happened across the lifespan, including in family contexts such as sibling bullying [4]. Bullying is a form of aggressive behavior that can negatively impact victims’ physical, emotional, and academic development [5]. It is an unwanted aggressive behavior, since it denotes a power imbalance between two or more people. This power imbalance includes differences in size or strength, popularity, abilities, or numbers [6]. In this respect, three characteristics must be present for the behavior to be considered bullying; the purpose of the behavior is to harm, the behavior repeatedly occurs over time, and in light of that, there is an imbalance of power [7]. Moreover, bullying can take different forms, including physical, verbal, and emotional [8]. Physical aggression, such as hitting, kicking, and shoving, is an overt physical action of bullying that falls under the category of physical bullying [9]. Verbal bullying includes verbal behaviors such as inappropriate language, nicknames, reprimanding, and verbal threats [10]. The hallmarks of emotional bullying typically include spreading false information, grouping up against others, ignoring, provocation, belittling, and humiliation [11]. Bullying takes place in a setting of power inequality, and it is goal-directed; therefore, it has the potential to cause serious injury [9].

Bullying is the most common form of aggression experienced by school-age youths [7], and it is a widespread social problem within schools, as it affects not only those who are subjected to bullying, but also students who bully others and witness bullying. Furthermore, it is considered a threat to mental health because it psychologically and physically affects the individual [12]. Bullying tends to increase during childhood and adolescence. However, when considering that late childhood and adolescence are times of rapid cognitive and socio-emotional development, questions remain about how bullying levels differ during these periods. For instance, Fujikawa et al. [13] aimed to investigate physical and verbal bullying, spreading rumors, social exclusion, cyberbullying, and multi-form bullying between grades 3 and 8. Bullying was prevalent in all of these grades, with 86% of children having reported it at least once in the previous four weeks at any wave, and 66% of them reported it frequently, while 37% of them reported it frequently in many forms. Teasing was the main frequent bullying type, whereas cyberbullying was less frequent. Moreover, whereas bullying reduced significantly for boys as they aged, it continued for girls into secondary school, with relational bullying predominating and cyberbullying increasing sharply in the early tens. Overall, bullying risks declined as students moved into secondary education.

A cross-sectional and cultural study by Biswas et al. [14] investigated the prevalence of bullying victimization among adolescent schoolchildren aged between 12 and 17 years in six World Health Organization (WHO) regions in 83 low-, middle-, and high-income countries between 2003 and 2015. The pooled prevalence of bullying victimization on one or more days in the previous 30 days was 30.5%. The highest prevalence was observed in the Eastern Mediterranean Region (45.1%, 44.3–46.0%) and the African region (43.5%, 43.0–44.3%) and the lowest in Europe (8.4%, 8.0–9.0%). Pooled prevalence among adolescents was lowest in high-income countries (20%, 19–20%) and greatest in the upper-middle-income group of low- and middle-income countries like Africa (40%, 40–41%). The study examined the cultural and social causes that are possibly related to the variation in frequency among males and females by location and country. For instance, bullying victimization rates were 45% for females and 42% for males in Africa, compared to 19% for females and 28% for males in Southeast Asia. According to this study, the majority of the countries lacked prospective follow-up information on bullying. Furthermore, higher levels of peer and parental support (e.g., understanding children’s problems and the importance of free time spent with children) were significantly associated with a reduced risk of bullying victimization. Therefore, parental and peer supports are protective factors against bullying victimization, and the reduction in bullying victimization may be facilitated by family- and peer-based interventions to increase adolescents’ social connectedness [14]. On the other hand, another study by Le et al. [15] found a correlation between bullying victimization and mental health problems, as bullying victimization is considered a predictor of subsequent mental health problems [12]. Victimization through bullying may worsen a person’s negative self-perception and make him feel more unsafe and threatened in his surroundings [16].

Violence in Algerian society continues to be a severe social evil and a complex reality that has attracted the attention of Algerian academics, researchers, and scholars, necessitating a review of both the country’s family and educational responsibilities and some suggested measures to lessen or end violence in schools [17]. Schools in Algeria continue to be riddled with violence. According to figures from the Ministry of Education, school violence has been classified as a “social earthquake”; more than 59,000 victims of violence, including teachers and students, were documented between 2001 and 2007 [18]. A high rate of bullying has been found in recent studies on the subject in Algerian schools. According to Fekih’s [19] research in 2018, bullying affects 10% to 16% of schools, with psychological bullying being the most prevalent type among the students. In a cross-sectional study involving 1452 students by Tiliouine [18], it was discovered that 15.1, 9.9, and 12.3% of students aged 8, 10, and 12 had experienced active bullying, while 16.3, 15.8, and 20.6% of those students had experienced passive bullying during the month prior to data collection. Additionally, the findings showed that children from less privileged homes and families that relocate to a new neighborhood are more likely to be the victims of bullying. Additionally, bullying victims are much more likely to miss class [18].

### 1.1. Sibling Bullying

Bullying can occur in different situations and environments. One potential place where bullying exists is the home environment with family members, specifically among siblings. The current literature shows a growing interest in understanding such sibling bullying (or sibling abuse) and its associated effects [20,21]. Historically, sibling abuse and peer bullying were considered “normal” adolescent rituals [22]. Although sibling aggression is relatively common, researchers are now considering whether some aggression can be considered as a form of bullying behavior [8].

Sibling bullying refers to any physical or verbal aggressive behavior involving one or both siblings. It includes hitting, pushing, kicking, spitting, biting, throwing objects, fighting, overplay, or name-calling [23]. Similar to traditional forms of bullying, sibling bullying involves an aggressive action which is often repeated over time [24]. Likewise, sibling bullying represents a serious and widespread problem, as Wolke and Skew [25] point out that the frequency of sibling bullying reaches 50% every month, and between 16% and 20% of them are involved in bullying several times a week. Wolke, Tippett, and Dantchev [23] found that up to 40% of siblings experience brotherly bullying every week, which is a frequent and harmful form of aggression in the family environment. According to Deniz et al. [26], 51% of Turkish adolescents (N = 301) aged from 10 to 18 stated that they had been victims of sibling bullying in the six prior months. Participants in the study of Peng et al. [27] also revealed that 12.5% of them had experienced bullying from their siblings, and 10.1% of them had experienced peer victimization, while 4.7% of them had experienced both sibling and peer victimization. Besides the high prevalence, exposure to sibling bullying in early childhood may lead to depression and self-harm problems, especially in early adulthood [28]. There is another piece of evidence that sibling bullying is linked to poor mental health [29], enduring reductions in mental health [30], depression and anxiety [27], and delinquent behaviors [31]. According to Wolke and Skew [25], the experience of sibling bullying increases the risk of engaging in school bullying, and this calls for studying the relationship between sibling bullying and school peer bullying.

### 1.2. Parental Acceptation–Rejection

In addition to sibling bullying, children’s interaction and relationship with their parents are important factors for their proper behavior and mental health. According to Rohner [32], one of the critical parental elements impacting children’s mental health is the acceptance–rejection factor [33]. The theory primarily examines parental love, including its expressions, effects, and sources [34]. Parental acceptance and rejection have been viewed as two poles of a continuum, with acceptance defining one end and rejection defining the other end. Four approaches, namely coldness/lack of affection (the reverse of warmth and affection), hostility/aggression, indifference/neglect, and undifferentiated rejection, can be used to indicate parental rejection [35]. According to the research of Mendo-Lázaro et al. [36], there is a direct link between emotional instability and criticism and rejection from parents. The relationship between children’s and adolescents’ emotional adjustment and family dynamics was confirmed, and in particular, maternal criticism and rejection in early adolescence and paternal criticism and rejection in middle adolescence were associated with emotional instability. In a longitudinal multicultural study [37], all four types of maternal and paternal rejection (coldness/lack of affection, hostility/aggression, indifference/neglect, and undifferentiated rejection) were found to be independently linked to both externalizing and internalizing problems among children and adolescents aged between 7 and 14 years old. The type of sibling bullying is also influenced by a number of important factors, including parents’ apparent acceptance or rejection. For instance, Kim and Kim [38] indicated that rejecting/neglecting parenting indirectly influenced peer victimization through sibling victimization. Kandemir Özdinç [35] also revealed that children’s perception of parental rejection was higher than their degrees of moral disengagement. On the other hand, lower levels of empathic propensity and problem-solving abilities led to higher levels of peer and sibling bullying behaviors.

### 1.3. Bullying between Siblings, Rejection by Parents, and Bullying at School

In most cases, similar violent behaviors are extended into multiple environments in a child’s life, such as home and school. In contrast, a home bully may be a school bully, which can be explained by personality traits, individual temperaments, family conflicts, and parental bullying [39]. In addition, relationships that bind family members and those outside the family come from several theoretical approaches. Social Learning Theory has suggested that children learn certain behaviors as a result of their relationships with their parents and siblings. Furthermore, these behaviors have been generalized in their interactions with colleagues and friends. The Theory of Attachment also suggests that children’s relationships with peers and siblings are affected by internal working models of relationships that are transferred from their early relationships with attachment models. Another process has linked family relationships to relationships outside the family, which is that the permanent characteristics of children, such as temperament, elicit similar responses from different partners in the relationship. Each of these theories indicates the existence of links between the children’s relationships with siblings, friends, and peers [25].

Sibling and peer relationships appear prominently in the daily experiences of children and adolescents as essential contexts for individual development. The experiences of children and adolescents in these relationships do not occur in isolation from each other, but rather they create vital links between these two crucial relationships in the process of development [40]. In a study by Johnson et al. [41], it was found that students who experienced bullying from siblings were more likely to suffer from school peer bullying. In comparison, Tippett and Wolke [42] found that sibling bullying increases the likelihood of being bullied by peers and is linked to concurrent and early adult mental problems, such as distress, depression, and self-harm. The study of Duncan [43] examined the prevalence of bullying among 375 children and identified a relationship between peer bullying and sibling bullying. The study revealed that 25% of these children reported being victims of bullying among their peers, and 28% of them admitted they were bullies. Hence, children who were bullied and victims of peer bullying reported the highest rate of bullying and abuse of siblings. Moreover, Morrill, Bachman, Polisuk, Kostelyk, and Wilson [22] found a relationship between surviving sibling abuse and peer bullying as well as between sibling bullying and school bullying. In a comparative study between sibling bullying and peer bullying [44], a sample of 392 young men answered questions about their experiences with sibling and peer bullying. The results demonstrated that participants perceived bullying behaviors between peers and siblings as somehow similar. While sibling bullying behaviors are reported and experienced more often compared to peer bullying behaviors, these studies provide so much evidence that there is a link between sibling bullying and peer bullying in school and that sibling bullying contributes to an increased risk of bullying among peers. It is also evidenced that those participants who reported sibling bullying were shown to be at an increased risk of peer bullying. According to Dantchev et al. [45], those children bullied at home and in school have no safe place to escape bullying harm.

Bullying also harms the social and emotional well-being of the child and adolescent, regardless of the perpetrator, and the child’s exposure to bullying by siblings is linked to similar results as school bullying. The study by Coyle et al. [46] aimed to examine the associations between peer and sibling bullying, internalizing behaviors, and the role of peer and sibling social support in relation to social and emotional well-being. The data were collected from students’ experiences of peer and sibling bullying and perceptions of social support among a sample of 372 primary school students. The results indicated that sibling and peer bullying were significantly associated with internalizing problems. In addition, peer social support reduced the relationship between sibling bullying and depression and reduced sibling social support between peer bullying and social pressure.

Indeed, acceptance and rejection by parents play an important role in school bullying and victimization. Mendo-Lázaro et al. [36] showed a clear association between maternal/paternal criticism and rejection with emotional instability in adolescence. Moreover, the study of Stavrinides et al. [47] showed that parental rejection predicted significant victimization, but parental rejection was not a significant predictor of bullying. Likewise, the results reported by Naseem [48] revealed that the direct effect of maternal acceptance–rejection was found as a significant and positive predictor of rejection sensitivity. In contrast, rejection sensitivity was found to be a significant positive predictor of bullying. In a study by Chen et al. [49], it was found that family climate has an indirect link with school bullying victimization through relationship with peers. Moreover, a study by Papadaki and Giovazolias [50] indicated that depressive symptoms moderated the association between victimization or bullying and parental rejection

To the best of the authors’ knowledge, family violence in general, specifically childhood offensive behavior, has received much attention over the past several decades, which professionals recognize as a large and widespread problem with lifelong consequences [51,52,53]. However, empirical evidence on the association between sibling victimization and bullying and school victimization and bullying is scarce. Moreover, despite being the most common forms of abuse within family systems, sibling victimization and bullying have been largely ignored in the literature. The present paper reports a field study aiming to investigate these concepts on middle school students in Algeria. Specifically, the study addresses the following research questions:

1. Does sibling victimization (in physical, emotional, and verbal forms) have an association with school victimization?
2. Does sibling bullying (in physical, emotional, and verbal forms) have an association with school bullying?
3. Does parental acceptance–rejection have an association with school victimization?
4. Does parental acceptance–rejection have an association with school bullying?

## 2. Materials and Methods

### 2.1. Research Design and Sample

A cross-sectional study was conducted among 221 students in middle schools at the Provinces of Chlef and Ouargla, Algeria. The inclusion criteria of the participants were (i) adolescents aged between 11 and 20 years old (ii) having at least a sibling. The participants’ mean age was 14.84 years (SD = 1.90). The majority of the participants, 148 (67%), were females, while 73 (33%) of them were males. The participants who were early adolescence (11 to 14 years) were 39.4% (n = 87); middle adolescence (14 to 18 years) was 58.8% (n = 130); late adolescence (18 to 20 years) represented 1.8% (n = 4). Concerning the number of siblings, the highest percentage (44.8%, n = 99) reported was four to six siblings, followed by 32.1% (n = 71) of the participants who had one to three siblings, while 23.1% (n = 51) of them had more than six siblings. Furthermore, the majority of adolescents reported that their parents were still living together (n = 197, 89.1%), followed by 4.1% (n = 9) who stated they had divorced parents; 6.8% (n = 15) reported a deceased parent (See Table 1 for participant characteristics).

### 2.2. Measures

#### 2.2.1. Sibling Bullying Scale (SBS)

The Sibling Bullying Scale (SBS) was developed by Aiche Sabah (2021). It consists of 26 items that measure two aspects of bullying: a bully and victim siblings. It measures three types of bullying and victimization, which are physical, emotional, and verbal bullying and victimization. The SBS items were reported using a five-point Likert scale: always = 5, never = 1 [54]. The psychometric properties of the SBS from the current study were satisfactory: internal consistency = 0.844 (bullying subscale) and 0.844 (victimization subscale); factor loadings = 0.429 to 0.816 (victimization subscale) and 0.371 to 0.874 (bullying subscale). The CFA of the SBS was supported by the fit indices in the confirmatory factor analysis (CFA): chi-square/df (bullying subscale= 2.284 and victimization subscale = 2.552), standardized root means square residual (SRMR) (bullying subscale = 0.0561 and victimization subscale = 0.0636), comparative fit index (CFI) (bullying subscale = 0.934 and victimization subscale = 0.914), and root mean square residual of approximation (RMSEA) (bullying subscale = 0.076 and victimization subscale = 0.084). The three dimensions (verbal, emotional, and physical) in the SBS had good internal consistency with maximal reliability (bullying subscale = 0.814, 0.758, 0.770 and victimization subscale = 0.764, 0.758, 0.770) and composite reliability (bullying subscale = 0.769, 0.752, 0.758 and victimization subscale = 0.727, 0.752, 0.758).

#### 2.2.2. Student Survey of Bullying Behavior—Revised 2 (SSBB-2)

The Student Survey of Bullying Behavior—Revised 2 (SSBB-2) in the current study used two subscales (victimization and bullying) of the SSBB-2 developed by Varjas et al. [55] to investigate students’ involvement in bullying behaviors at school. These two subscales of the questionnaire, victimization and bullying, are the focus of the current study. The scale includes 24 items: 12 items regarding the number of times a student was the target of bullying and 12 items regarding a student’s bullying of other students. The SSBB-2 items were responded to using a five-point Likert scale: always = 5, not at all = 1. Prior research showed that the SSBB-2 has satisfactory internal consistency, with 0.86 in the bullying subscale and 0.93 in the victimization subscale [56].

The psychometric properties of the SSBB-2 from the current study were satisfactory. The three-factor structure of the SSBB-2 victimization subscale was supported by the fit indices in the CFA: chi-square/df = 1.658, SRMR = 0.063, comparative fit index (CFI) = 0.954, and RMSEA = 0.055. The three dimensions (verbal, emotional, and physical) in the SSBB-2 victimization subscale had good internal consistency, with maximal reliability = 0.712, 0.741, and 0.732 and composite reliability = 0.677, 0.712, and 0.702.

The three-factor structure of the SSBB-2 bullying subscale was supported by the fit indices in the CFA: chi-square/df = 2.788, SRMR = 0.057, CFI = 0.93, and RMSEA = 0.09. The three dimensions (verbal, emotional, and physical) in the SSBB-2 victimization subscale had good internal consistency, with maximal reliability = 0.819, 0.804, and 0.770 and composite reliability = 0.795, 0.775, and 0.760.

#### 2.2.3. Parental Acceptance–Rejection Questionnaire (PARQ)

The perception of parental acceptance and rejection questionnaire was adopted from Erraji [57]. It consists of 12 items: six items to measure parental acceptance and six to measure parental rejection. The response was given by a four-point scale: occasionally = 1, always = 4. Concerning the scale’s validity, Erraji [57] found that the correlations between the items ranged from 0.73 to 0.91, and another study by Abdullah and Aziz [58] reported good psychometric properties. Furthermore, the psychometric properties of the PARQ from the current study were satisfactory. The two-factor structure of the PARQ was supported by good fit indices in the CFA analysis, with chi-square/df = 1.934, SRMR = 0.077, comparative fit index (CFI) = 0.916, and RMSEA = 0.065. The two dimensions (parental acceptance and parental rejection) in the PARQ victimization subscale had good internal consistency, with maximal reliability = 0.747 and 0.825 and composite reliability = 0.734 and 0.771.

### 2.3. Statistical Analyses

IBM SPSS Statistics, Version 28 (IBM Corporation, North Castle Drive, MD-NC119 Armonk, NY 10504-1785, US) was used for descriptive statistics in this study, while AMOS Version 24 (IBM Corporation, North Castle Drive, MD-NC119 Armonk, NY 10504-1785, US) was used for structural equation modeling (SEM). The SEM instead of the multiple linear regression model was used in the present study because the SEM has the strengths of simultaneously taking measurement errors into account when assessing the associations. Although regression models can also be used to assess the associations, they do not consider measurement errors [59]. Moreover, SEM allows for simultaneously estimating a series, but independent, multiple regression equations cannot. It can also incorporate latent variables into the analysis and accounts for measurement errors in the estimation process [60,61,62,63,64]. In other words, SEM is a statistical technique that establishes measurement models and structural models to address complicated behavioral relationships [61,62,65,66]. Therefore, SEM with the maximum likelihood estimator was used to explore the association between sibling bullying through its two subscales (the victim and the bully) and school bullying (the bullying student and the victim) and the association between parental acceptance–rejection and school bullying. Fit indices were used to examine the psychometric properties for the measurements used in the current study and to evaluate the fits of the models testing the associations between study variables. SEM was evaluated using several goodness-of-fit indices. More specifically, the model fit was evaluated using fit indices of nonsignificant χ2, comparative fit index (CFI) > 0.90, together with standardized root mean square residual (SRMR) and the root mean square error of approximation (RMSEA) < 0.08 [66,67,68,69,70,71]. Furthermore, maximal and composite reliability were used to estimate the model’s validity. A composite reliability and maximal reliability of 0.7 or higher are considered good, and between 0.6 and 0.7 is acceptable [72,73].

## 3. Results

### 3.1. Association between Sibling Victimization and School Victimization

**Figure 1:**
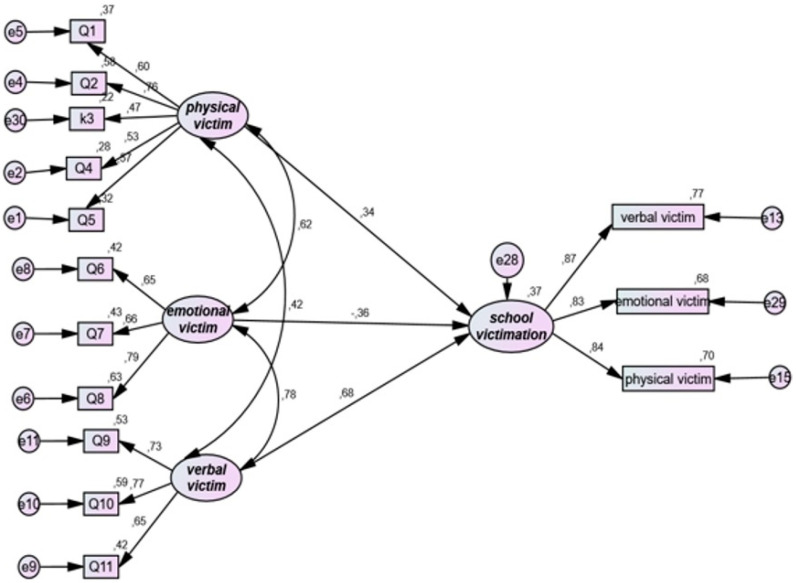
Structural model for research question 1. Note the structural equation modeling links between physical, emotional, and verbal bullying practiced against the brothers (the victim) as an independent variable and school victimization as a dependent variable.

Table 2 and Figure 1 display the associations between sibling victimization and school victimization. The SEM fit indices indicate an acceptable fit for the model testing the association between sibling victimization and school victimization: chi-square = 185.718; chi-square/df = 2.616; CFI = 0.902; SRMR = 0.068; RMSEA = 0.086. Moreover, the results showed that sibling physical victim was significantly associated with school victimization (standardized coefficient = 0.341; *p* = 0.006), whereas sibling emotional victim was not significantly associated with school victimization (standardized coefficient = −0.357; *p* = 0.111); sibling verbal victim was significantly associated with school victimization (standardized coefficient = 0.682; *p* < 0.001).

### 3.2. Association between Sibling Bullying and School Bullying

**Figure 2:**
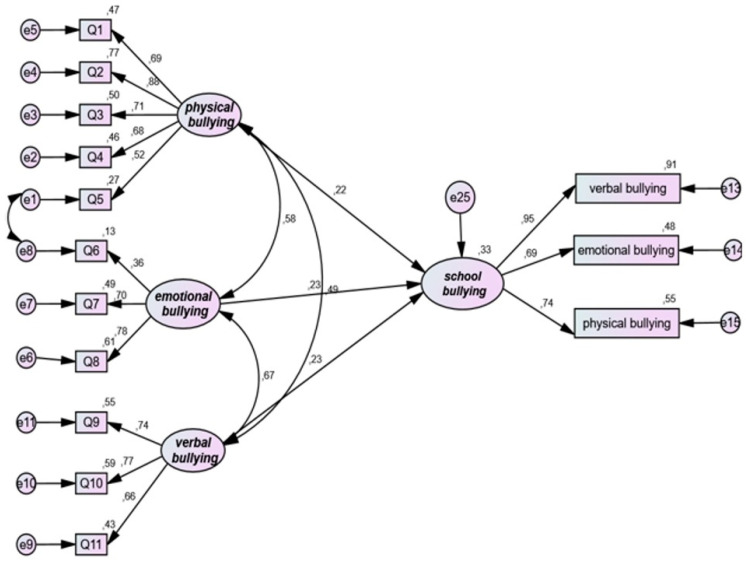
Structural model for research question 2. Note the structural equation modeling links between physical, emotional, and verbal bullying by brothers (the bullying) as an independent variable and school bullying as a dependent variable.

Table 3 and Figure 2 display sibling and school bullying associations. The SEM fit indices indicate an acceptable fit for the model testing the association between sibling bullying and school bullying: chi-square = 104.957; chi-square/df = 1.499; CFI = 0.970; SRMR = 0.055; RMSEA = 0.048. Moreover, the results showed that sibling physical bullying was significantly associated with school bullying (standardized coefficient = 0.219; *p* = 0.016), while sibling emotional bullying was not significantly associated with school bullying (standardized coefficient = 0.228; *p* = 0.077); sibling verbal bullying was significantly associated with school bullying (standardized coefficient = 0.232; *p* = 0.041).

### 3.3. Association between Parental Acceptance and Rejection and School Victimization

**Figure 3:**
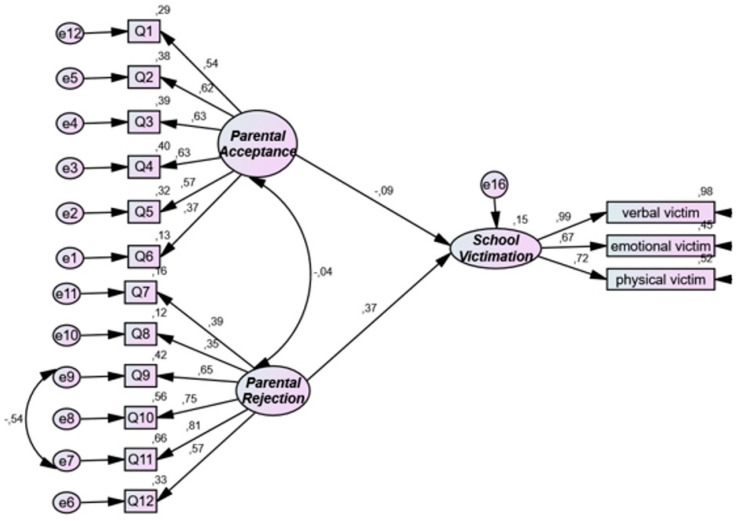
Structural model for research question 3. Note the structural equation modeling links between parental acceptance and rejection as an independent variable and school victimization as a dependent variable.

Table 4 and Figure 3 display the associations between parental acceptance/rejection of adolescents and school victimization. The SEM fit indices indicate an acceptable fit for the model testing the association between parental acceptance/rejection of adolescents and school victimization: chi-square = 160.051; chi-square/df = 1.861; CFI = 0.915; SRMR = 0.078; RMSEA = 0.063. Moreover, the results showed that parental acceptance was not significantly associated with school victimization (standardized coefficient = −0.092; *p* = 0.228); parental rejection of adolescents was significantly associated with school victimization (standardized coefficient = 0.371; *p* < 0.001).

### 3.4. Association between Parental Acceptance and Rejection and School Bullying

**Figure 4:**
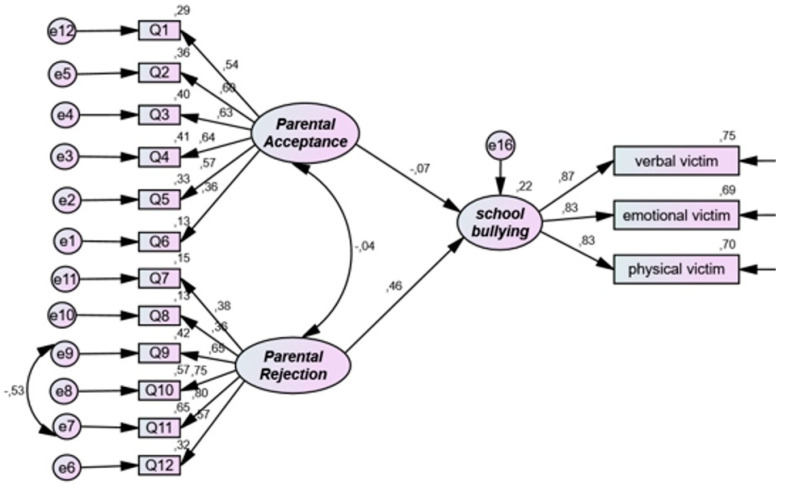
Structural model for research question 4. Note the structural equation modeling links between parental rejection of adolescents as an independent variable and school bullying as a dependent variable.

Table 5 and Figure 4 display the associations between parental acceptance/rejection of adolescents and school bullying. The SEM fit indices indicate an acceptable fit for the model testing the association between parental acceptance/rejection of adolescents and school bullying: chi-square = 159.545; chi-square/df = 1.855; CFI = 0.923; SRMR = 0.076; RMSEA = 0.062. Moreover, the results showed that parental acceptance was not significantly associated with school bullying (standardized coefficient = −0.070; *p* = 0.369); parental rejection of adolescents was significantly associated with school bullying (standardized coefficient = 0.464; *p* < 0.001).

## 4. Discussion

Using cross-sectional data on adolescent students in the school context of Algeria, this study aimed to determine the contribution of sibling bullying in its two forms: bullies of sibling bullying and victims of sibling bullying in the interpretation of school bullying victimization among peers in a sample of school-age adolescents. Moreover, the study investigated the effect of parental acceptance or rejection on school bullying victimization. The main findings of the present study are (i) sibling bullying was associated with school bullying and school victimization, (ii) sibling bullying and parental acceptance/rejection were associated with school bullying, (iii) sibling emotional bullying and sibling emotional victims were not associated with school bullying or victimization, and (iv) parental rejection was associated with school victimization and school bullying. 

The findings demonstrated that any experience of sibling bullying (either victim or bully) is associated with school bullying and school victimization. In particular, sibling physical victims and sibling verbal victims appeared to influence school victimization, but the effect of sibling emotional victims on school victimization was not statistically significant. The results showed that sibling physical bullying and sibling verbal bullying had effects on school bullying. As to the effect of sibling emotional bullying on school bullying, it was not statistically significant. These results suggest a high impact of being consistently bullied (i.e., a victim) by siblings on school bullying.

Sibling bullying and parental acceptance/rejection contribute to school bullying. This finding is consistent with previous research in this area [27,45,46,48]. In reality, family factors contribute to sibling bullying, as Wolke, Tippett, and Dantchev [23] believe that family characteristics have some effect on bullying rates because they represent the primary environment in which siblings interact. After reviewing previous studies on sibling bullying, Wolke, Tippett, and Dantchev [23] classified family characteristics that contribute to bullying in general into three categories: structural factors (e.g., family formation, number of siblings, birth order, and gender of siblings), socio-economic factors (e.g., family income, parenting education, and profession), and adult or caregiver behavior (e.g., child maltreatment and parenting behavior).

As for sibling emotional bullying and sibling emotional victims, it was found that there were no statistically significant associations with school bullying or victimization. The results of the present study support previous studies. Relevant literature indicates that adolescence sees a decline in emotional involvement and intimacy with siblings [20,21]. In addition, adolescents spend less time with their siblings. These developmental trends are invariably linked to youth’s increasing autonomy in other aspects of their lives, such as increased interaction with friends, classmates, and romantic partners, as well as their recreational hobbies [20,21].

The associations between parental acceptance and school bullying/victimization were not statistically significant. In contrast, parental rejection was significantly associated with both school victimization and school bullying. These results are supported by the Theory of Parental Acceptance–Rejection [32] as cited in Papadaki and Giovazolias’ work [50], according to which parental rejection has been shown to be a major cause of emotional, behavioral, cognitive, and social development problems for children. Likewise, this finding corroborates the results of previous studies [47,48].

### 4.1. Research Implications 

In this study, we have sought to understand bullying from a family to school perspective. We have researched, in particular, the effects of sibling bullying and parental acceptance on school bullying. The findings provide theoretical and practical implications for the field of family psychology and adolescents in general.

Our findings help to address the lack of studies in the Arab environment on fraternal bullying and its role in school bullying, as we have noticed that many studies treat school bullying, but such studies ignore its relationship to bullying in the family. Accordingly, the first major practical contribution of the present research is that it provides empirical data about the effect of family factors (sibling bullying and parental acceptance) on school bullying. This finding is important given the spread of bullying in schools; therefore, it is important to identify its true causes in order to reduce this dangerous phenomenon among adolescents.

### 4.2. Program/Policy Implications

This study is an important addition to the program/policy implications regarding bullying reduction. Specifically, the present findings in the family and the school are in agreement with the previous literature. Therefore, our findings echoed the importance of taking care of aggressive behaviors and promoting warmth in both the family and school contexts. In other words, programs and policies are needed to take care of bullying reduction to avoid the possibility that children learn bullying behaviors in the family and generalize them to their interactions and social relationship with peers.

### 4.3. Future Directions

According to the present study’s findings, future studies may want to design effective programs to reduce school and sibling bullying. Therefore, studies are also needed to evaluate if there are effective programs for bullying reduction. For example, future studies may want to examine if promoting family warmth (e.g., increasing parental acceptance and decreasing parent rejection) is helpful for reducing bullying. In addition, future studies may want to explore if the present study’s findings can be generalized to other cultural contexts (e.g., Western countries), given that bullying is an issue associated with cultures.

### 4.4. Limitations

These findings provide preliminary evidence for the contribution of sibling bullying and parental acceptance to school bullying, but such results cannot be generalized to adolescents around the world, because many considerations restrict such generalizations. The first limitation is that the study sample was selected using a cross-sectional sampling method. Longitudinal studies, therefore, should be conducted in order to provide more reliable results. The second limitation is related to the method of collecting data that relies on psychological scales, which only express the adolescents’ point of view and may be biased, as other data collection tools, such as interviews, may give more accurate results. The third constraint is the sample size, which was far lower than the study population. The study was conducted only on a sample from the state of Chlef in Algeria due to the difficulty of including all Algerian school students in the study.

## 5. Conclusions

The results of the present study showed a statistically significant effect of sibling bullying/victimization on school bullying/victimization. Moreover, parental rejection was found to affect school bullying/ victimization significantly. These results offer theoretical implications for the literature on how to address bullying among adolescence with families’ and siblings’ contributions to school bullying. Specifically, our study proposes that the family context plays a fundamental role in school bullying. These findings can help researchers, teachers, educators, and parents better understand the important family-related factors that lead to students’ school bullying.

## Figures and Tables

**Table 1:** Participant properties.

Variables	Groups	Frequency	Percentage
Gender	Boys	73	33.0
	Girls	148	67.0
Stages of adolescence	Early adolescence (11–14 years)	87	39.4
	Middle adolescence (14–18 years)	130	58.8
	Late adolescence (18–20 years)	4	1.8
Number of siblings	1 to 3	71	32.1
	4 to 6	99	44.8
	More than 6	51	23.1
Parental status	Live together	197	89.1
	Divorced	9	4.1
	One deceased	15	6.8

**Table 2:** Association of sibling physical, emotional, and verbal victimization with school victimization.

Variables			Regression Weights				Standardized Regression Weights
			Estimate	S.E.	t	P	Estimate
School victimization	<---	Sibling physical victim	1.287	0.473	2.724	0.006	0.341
School victimization	<---	Sibling emotional victim	−1.194	0.750	−1.592	0.111	−0.357
School victimization	<---	Sibling verbal victim	2.599	0.748	3.475	<0.001	0.682

**Table 3:** Estimates of the effect of the physical, emotional, and verbal bullying by brothers (the bullying) on school bullying.

Variables			Regression Weights				Standardized Regression Weights
			Estimate	S.E.	t.	P	Estimate
School bullying	<---	Sibling Physical bullying	0.954	0.398	2.398	0.016	0.219
School bullying	<---	Sibling emotional bullying	0.821	0.464	1.771	0.077	0.228
School bullying	<---	Sibling verbal bullying	0.967	0.472	2.047	0.041	0.232

**Table 4:** Estimates of the effect of parental acceptance and rejection of adolescents on school victimization.

Variables			Regression Weights				Standardized Regression Weights
			Estimate	S.E.	t	P	Estimate
School victimization	<---	Parental Acceptance	−0.678	0.562	−1.205	0.228	−0.092
School victimization	<---	Parental Rejection	2.004	0.419	4.782	***	0.371

*** = *p* < 0.001

**Table 5:** Estimates of the effect of parental acceptance and rejection on school bullying.

Variables			Regression Weights				Standardized Regression Weights
			Estimate	S.E.	t	P	Estimate
School bullying	<---	Parental acceptance	−0.383	0.426	−0.898	0.369	−0.070
School bullying	<---	Parental rejection	1.863	0.343	5.438	***	0.464

*** = *p* < 0.001

## Data Availability

The dataset that supports the findings of this study is not openly available and it will be available from the corresponding author upon reasonable request.
